# Privacy Preserving Technologies in US Education.

**DOI:** 10.23889/ijpds.v7i3.2084

**Published:** 2022-08-25

**Authors:** Amy O'Hara, Stephanie Straus

**Affiliations:** 1 Georgetown University

In the US education sector, data are captured on learners at all stages of the life course with rich, sensitive information on learner demographics, enrollment, achievement, borrowing, and outcomes. Most data are controlled by institutions, who increasingly want to monitor progress from cradle to career, tracking students from early childhood through primary and secondary schools (K-12), on to degrees granted in postsecondary institutions, including continuing and technical education that grant credentials. Collecting, storing, and using such data raises privacy concerns.

This presentation describes privacy preserving technologies (PPTs) that have been discussed, tested, implemented – and abandoned – in the education domain. These include both input and output privacy protection methods ranging from absolute lockdowns of learner data to cryptographically-protected data access and analysis systems. We describe most familiar and tested PPTs including secure hashing, secure multiparty computation, trusted execution environments, and differential privacy, highlighting actual applications in schools, departments of education, and educational technology nonprofits and companies.

We summarize the factors that limit PPT use and success, including legal, institutional, technical, and cultural barriers and offer recommendations to overcome these barriers. For example, we find gaps in understanding what PPTs are and what problems they solve; a lack of engagement with data controllers, subjects, and regulators; and a lack of human capital to implement and use PPTs. There are also significant research and development needs to continue making PPTs less costly in financial, compute, and complexity terms. Our recommendations suggest topics for continued research into methods that produce disaggregate statistics to analyze equity while protecting privacy, have clear and fair privacy loss budgets, and produce open-source technology, ideally pairing scientists with education experts.

